# Can activities of daily living contribute to EMG normalization for gait analysis?

**DOI:** 10.1371/journal.pone.0174670

**Published:** 2017-04-03

**Authors:** Aseel Ghazwan, Sarah M. Forrest, Cathy A. Holt, Gemma M. Whatling

**Affiliations:** 1 Cardiff School of Engineering, Cardiff University, Cardiff, United Kingdom; 2 Arthritis Research UK Biomechanics and Bioengineering Centre, Cardiff University, Cardiff, United Kingdom; University of Toronto, CANADA

## Abstract

This study aims to examine alternative methods of normalization that effectively reflect muscle activity as compared to Maximal Voluntary Contraction (MVC). EMG data recorded from knee flexion-extension muscles in 10 control subjects during the stance phase of the gait cycle were examined by adopting different approaches of normalization: MVC, Mean and Peak Dynamic during gait cycles, (MDM and PDM, respectively), Peak Dynamic during activities of daily living (ADLs), (*PDM), and a combination of ADLs and MVC(**PDM). Intra- and inter-individual variability were calculated to determine reliability and similarity to MCV. **PDM showed excellent reliability across subjects in comparison to MVC, where variance ratio ranged from 0.43–0.99 for **PDM and 0.79–1.08 for MVC. Coefficient of variability showed a similar trend to Variance Ratio, ranging from 0.60–1.25 for **PDM and 1.97–3.92 for MVC. Both MVC and **PDM, and to some extent *PDM, demonstrated good-to-excellent relative amplitude’s matching; i.e. root mean square difference and absolute difference were both around 0.08 for Vastus medialis to about 4 for Medial gastrocnemius. It was concluded that **PDM and *PDM were reliable, **PDM mirrored MVC and thus could be used as an alternative to MVC for subjects who are unable to provide the required effort for MVC testing. Where MVC testing is not possible, *PDM is the next preferred option.

## 1. Introduction

Surface electromyography (EMG) has been used for decades to evaluate neuromuscular responses during a range of activities, in non-pathological subjects [1, 2] and to investigate alterations due to pathology [3] and rehabilitation. The EMG signal amplitude and frequency components are affected by many factors: electrode placement and orientation [4], subcutaneous fat thickness, muscle fiber type, and crosstalk from nearby muscles [5]. Normalization of the EMG signal reduces the negative impact of the aforementioned variables and facilitates the comparison of EMG across muscles, between subjects, or between days for the same subject [6–9].

Different methods of normalization have been introduced and are in current practice, each with advantages and limitations related to inter-subject variability and the clinical interpretation of muscle activity. The peak dynamic method (PDM) [10] expresses EMG data from a muscle as a ratio of the peak value acquired from the same muscle during gait. For the mean dynamic method (MDM), each data-point of the processed EMG signal is divided by an average of both quiet and active periods during the gait cycle. Both PDM and MDM produce good reliability between sessions and between subjects [11–13], reduce inter-subject variability by 12–72% [11] in comparison to un-normalized EMG and give a representation of coordinated muscle activity [3] by indicating the period at which the muscle is most active [14]. The third method of normalization [15] is maximum voluntary contraction (MVC). Each data point is divided by the peak value recorded from an isometric maximal voluntary contraction of the same muscle. The reference value used for normalization (the denominator), called the normalization factor [16], should be processed in an identical manner as the task EMG. Although EMGs normalized by MVC have been shown to display poor reliability [6, 17, 18], MVC is highly recommended by many reported studies as it reflects a true increase or decrease in the neural drive, if it is performed under isometric conditions [6, 13, 14]. It is important for gait analysis studies to understand neuromuscular activation and how it contributes to external forces to stabilize joints and distribute joint loading.

The question “What is the best method of normalization?” initiated an ongoing discussion at the beginning of the eighties. Researchers attempted to answer this question by taking into account that the reference value should be reliable across time and between subjects. It should also be meaningful by reflecting the muscle activity and timing of activation.

Patients with compromised function due to disease or injury and in some cases, healthy control subjects, are unable to maximally contract their muscles during MVC tasks[11]. The current study therefore examines if there is an alternative method that is comparable to the MVC in reflecting muscle activity for level gait, whilst having good repeatability.

## 2. Materials and methods

### 2.1 Experimental data

Three-dimensional kinematic and kinetic data of ten healthy subjects were collected. The mean and standard deviation (SD) of age, mass and height were 30.6(3.8) years, 78.04 (10.95) kg, and 1.74 (0.04) m respectively.

All subjects gave their informed written consent prior to data collection. The consent and data protocols were approved by the Research Ethics Committee for Wales and Cardiff and Vale University Health Board.

Experimental gait data was collected in the motion analysis laboratory at Cardiff School of Engineering. Gait analysis was performed using nine 120Hz infra-red motion capture units (Qualisys, Sweden). Qualisys Track Manager (QTM, Qualisys, Sweden) was used to capture full body motion using reflective markers placed on the trunk, pelvis, and both the upper and lower limbs (modified Cleveland clinic marker set)[19]. Four floor-embedded force platforms (Bertec Corporation) were used to capture the ground reaction force vectors with a sample rate of 1080 Hz.

Muscle electromyographic (EMG) data were collected bilaterally, using Trigno™ Wireless EMG System (Delsys, Inc.), for seven muscles: Rectus Femoris, Vastus Lateralis, Vastus Medialis, Biceps Femoris, Semitendinosus, Gastrocnemius Lateralis, and Gastrocnemius Medialis. The electrodes were placed longitudinally over the muscle bellies after standard preparation of the skin, according to SENIAM recommendations [20], involving shaving, exfoliation, cleaning of the skin and finally electrode gel was used to reduce the electrode–skin impedance [21].

MVC for all muscles were measured first. Three tests were performed to maximally activate knee muscles:

- Isometric MVC for Quadriceps: Each subject sat on a chair with their hips flexed to 90° and their knee fully extended, they were asked to resist a force being applied downwards on the extended leg at the level of ankle joint.
- Isometric MVC for Hamstring: In a standing position, with one knee flexed to 90°, the subject was asked to resist a force applied downwards on the flexed leg at the level of ankle joint.
- Isometric MVC for Gastrocnemii: subjects were instructed to stand on toe-tips and maximally contract their shank muscles as much as possible. Visual feedback of the EMG signal and strong verbal encouragement [22] was provided.

Then individuals were asked to perform different ADLs (i.e., walking at their comfortable speed, walking up and down stairs and finally sitting-to-standing). Meanwhile, information regarding muscle EMG, ground reaction force and three dimensional movements were collected using the synchronized movement analysis system. 6 trials of level gait, 6 trials of ascending/descending a four step staircase, and 2 trials of standing/sitting were recorded for each subject.

The stance phase was determined by the ground reaction force measured by the force plate, at heel strike to toe-off.

### 2.2 EMG signal processing

EMG data, through stance phase, were analysed in Matlab (version R2013a, Mathworks Inc.). The raw EMG signals were band-pass filtered, to remove the movement artefacts, by a Butterworth 4th order filter at (10_450) Hz, rectified and finally low-pass-filtered with a 4th order Butterworth filter at 6 Hz to create a linear envelope for each muscle. Then linear envelopes for each muscle were normalized by using:

1. MVC, each data point during stance phase was divided by the peak value recorded from an isometric maximal voluntary contraction of the same muscle.
2. PDM, each data point during stance phase was divided by the peak value for the muscle among six gait trials.
*PDM, each data point during stance phase was divided by the peak value for the muscle measured among six trials of level gait, ascending/descending stairs, and standing/sitting.
**PDM, each point during stance phase was divided by the peak value for the muscle acquired from all trials of gait level, ascending/descending stairs, standing/sitting, and MVC.
5. MDM, each data point during stance phase was divided by the mean value for the muscle from the six gait trials.

The EMG data were then normalized to 100 data points though the stance phase.

In order to determine which method of normalization provided the most repeatable data set, the variance ratio (VR) and coefficient of variation (CV) were calculated. Root Mean Square Difference (RMSD), Absolute Difference (ABSD), and Percentage Difference (%D) were measured to determine which normalization method more closely matched normalization by MVC.

**Fig 1:**
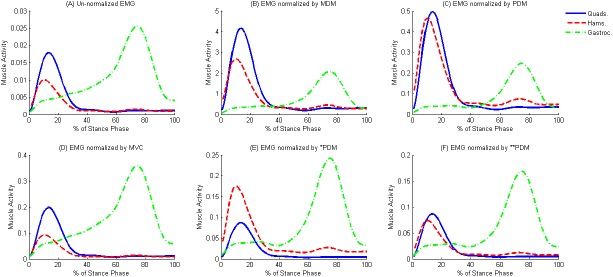
Muscle activity, for a healthy subject (mean for 6 trials) is expressed in percent Stance Phase for un-normalized EMG; (A), EMG normalized by MDM; (B), EMG normalized by PDM; (C), EMG normalized by MVC; (D), EMG normalized by *PDM;(E), and EMG normalized by **PDM; (F). Quads = quadriceps muscles, Hams = hamstring muscles, Gastroc = gastrocnemii muscles.

An average of six trials for each muscle and for each participant were created to represent the mean muscle activity through stance, (example given in Fig 1).

### 2.3 Assessment of inter-individual variability

#### 2.3.1 The variance ratio (VR)

Used to assess the inter-individual variability (i.e. reliability) of the non-normalized and normalized gait EMGs for each muscle [23].

$$VR=\frac{\sum_{i=1}^{k}\sum_{j=1}^{n}{\left(X_{ij}-{\bar{X}}_{i}\right)}^{2}/k(n-1)}{\sum_{i=1}^{k}\sum_{j=1}^{n}{\left(X_{ij}-\bar{X}\right)}^{2}/\left(kn-1\right)}$$ (1)

where, k is the number of time intervals over the gait cycle, n is the number of trials (i.e. 6) for intra-individual variability, or the number of participants (i.e. 10) for inter-individual variability, X_ij_ is the EMG value at the ith interval for the jth trial (intra-individual variability) or participant (inter-individual variability), and ${\bar{X}}_{i}$ is the mean of the EMG values at the ith time interval over j gait cycles (intra-individual variability) or participants (inter-individual variability), $\bar{X}$ is the mean.

$$\bar{X}=\frac{1}{k}\sum_{i=1}^{k}{\bar{X}}_{i}$$ (2)

#### 2.3.2 Coefficient of variation (CV)

Useful for comparing the degree of variation from one data series to another, even if the means are drastically different from each other.

$$CV=\frac{\sqrt{\frac{1}{k}\sum_{i=1}^{k}\sigma_{i}^{2}}}{\frac{1}{k}\sum_{i=1}^{k}\left|\bar{X_{i}}\right|}$$ (3)

where, k is the number of time intervals over the gait cycle, ${\bar{X}}_{i}$ is the mean of the EMG values at the ith interval calculated over six trials for intra-individual variability or 10 participants for inter-individual variability, σ_i_ is the standard deviation of the EMG values about ${\bar{X}}_{i}$ calculated over six trials for intra-individual variability or 10 participants for inter-individual variability.

#### 2.3.3 The Root Mean Square Difference (RMSD), the Absolute Difference (ABSD), and the Percentage Difference (%D)

Useful for comparing the differences between the MVC and the other methods of normalization.

A mean and SD was calculated for each of the three differences by including values from all gait cycles and for all participants.

$$RMSD=\sqrt{\frac{1}{k}\sum_{i=1}^{k}{\left(X_{ia}-X_{ib}\right)}^{2}}$$ (4)

$$ABSD=\frac{1}{k}\sum_{i=1}^{k}\left|X_{ia}-X_{ib}\right|$$ (5)

$$\%D=\frac{1}{k}\sum_{i=1}^{k}(\frac{\left|X_{ia}-X_{ib}\right|}{X_{ia}})\;100$$ (6)

where, k is the number of time intervals over the gait cycle, X_ia_ is the EMG value at the ith interval normalized using the isometric MVC method, and X_ib_ is the EMG value at the ith interval normalized using the other method of normalization.

### 2.4 Statistical analysis

Muscle activations during each stance phase of gait were normalized to percentage stance phase and then the mean and standard deviation calculated for each subject. 95% confidence intervals were calculated to determine if statistically significant differences existed between the un-normalized and normalized EMGs. A Kruskal Wallis test of nonparametric data was performed for each muscle in each subject using SPSS (version 20, Chicago, IL).Group means and standard deviations were calculated among subjects and figures were created using Matlab 3013a.

## 3. Results

To enable comparisons with previous literature, EMG patterns for the quadriceps, hamstrings, and gastrocnemii were plotted through stance phase (Fig 1). In agreement with [24], the hamstrings were predominantly activated in early stance followed by the quadriceps, to stabilize the knee joint during the load bearing period, whereas the gastrocnemii contributed to the tibiofemoral force in late stance.

Data across participants were consistent and one data set was illustrated as a representative example; i.e. (Fig 1A, 1B, 1C, 1D, 1E and 1F) shows the un-normalized EMG signals and those normalized by MDM, PDM, MVC, *PDM and **PDM, respectively for the individual during the stance phase (mean of 6 gait trials). While there were similarities between the activation patterns for the methods of normalization, in which hamstring is highly activated in early stance followed by quadriceps and finally by gastrocnemius, there were also profound differences in terms of relative amplitude. Results showed that the amplitude of the normalized muscle were significantly greater (p < 0.05) than the un-normalized for all methods of normalization.

**Fig 2:**
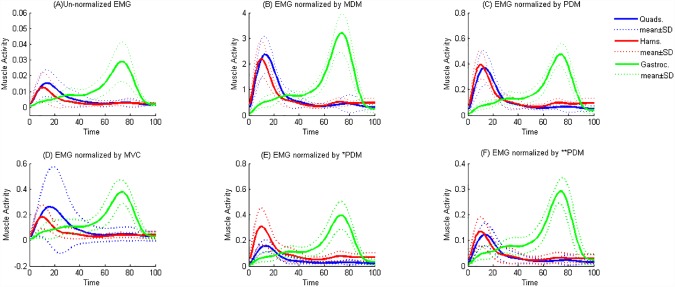
Muscle activity, for 10 healthy subjects is expressed in percent Stance Phase for un-normalized EMG; (A), EMG normalized by MDM; (B), EMG normalized by PDM; (C), EMG normalized by MVC; (D), EMG normalized by *PDM;(E), and EMG normalized by **PDM; (F). Quads = quadriceps muscles, Hams = hamstring muscles, Gastroc = gastrocnemii muscles, n = 10.

During ADLs, subjects adopt different strategies to muscle coordination. There is no clear trend regarding which activity gives the peak value for each muscle across subjects, with the exception for Vastii during ascending stairs; where the Vastus lateralis reached maximum values in 7 from 10 subjects and Vastus medialis reached maximum values in 6 from 10 subjects. The other muscles reached their maximum values through ascending stairs, in just 4 from 10 subjects.

(Fig 2A, 2B, 2C, 2D, 2E and 2F) shows the un-normalized EMG signals and those normalized by MDM, PDM, MVC, *PDM and **PDM, respectively for 10 healthy subjects during stance phase. Fig 2D illustrates a high standard deviation for the quads when using the MVC method of normalization, which demonstrates the potential negative impact of this method.

Tables 1, 2 and 3 summarize the reliability indices across trials and subjects; i.e., CV and VR, respectively. No significant differences were found for the inter-individual variability, VR and CV, of the un-normalized and normalized EMG. Tables 4 and 5 summarize descriptive statistics for RMSD, ABSD, and %D measured between the MVC and the other methods of normalization.

**Table 1:** Intra-individual variability (CV = Coefficient of Variation; VR = Variance Ratio) for un-normalized EMGs* during stance phase of gait cycle, n = 10.

		RF	VL	VM	BF	ST	LG	MG
**CV**	**mean**	0.53	0.43	0.42	0.48	0.43	0.40	0.31
	**SD**	0.52	0.16	0.17	0.16	0.12	0.11	0.06
**VR**	**mean**	0.34	0.18	0.22	0.30	0.29	0.23	0.17
	**SD**	0.28	0.12	0.14	0.18	0.09	0.15	0.07

RF, Rectus Femoris; VL, Vastus Lateralis; VM, Vastus Medialis; BF, Biceps Femoris; ST, Semitendinosus; LG, Lateral Gastrocnemius; ML, Medial Gastrocnemius.

*intra-individual variability were the same for the un-normalized EMGs and those normalized by the methods employed.

**Table 2:** Inter-individual variability (Coefficient of Variation; CV) for un-normalized EMGs and EMGs normalized by MDM, PDM, *PDM, and **PDM during stance phase of gait cycle, n = 10.

	RF	VL	VM	BF	ST	LG	MG
**Un-normalized**	1.13	1.68	0.84	0.84	1.02	0.53	0.67
**MVC**	1.97	3.54	1.16	3.64	3.92	3.43	3.87
**MDM**	0.63	0.57	0.56	0.48	0.53	0.48	0.59
**PDM**	0.77	0.57	0.65	0.51	0.54	0.51	0.61
*PDM	0.97	0.70	0.78	0.59	0.79	0.48	0.65
**PDM	1.60	0.93	0.94	0.89	1.25	0.60	0.71

RF, Rectus Femoris; VL, Vastus Lateralis; VM, Vastus Medialis; BF, Biceps Femoris; ST, Semitendinosus; LG, Lateral Gastrocnemius; ML, Medial Gastrocnemius

*PDM refers to the method of normalization by using the peak values through ADLs

**PDM refers to the method of normalization by using the peak values through ADLs and MVC.

**Table 3:** Inter-individual variability (Variance Ratio; VR) for un-normalized EMGs and EMGs normalized by MDM, PDM, *PDM, and **PDM during stance phase of gait cycle, n = 10.

	RF	VL	VM	BF	ST	LG	MG
**Un-normalized**	0.91	0.86	0.61	0.58	0.74	0.37	0.57
**MVC**	1.03	1.02	0.79	1.06	1.06	1.08	1.06
**MDM**	0.62	0.29	0.34	0.31	0.39	0.31	0.48
**PDM**	0.70	0.29	0.44	0.37	0.40	0.34	0.51
*PDM	0.83	0.39	0.56	0.43	0.63	0.32	0.55
**PDM	0.99	0.50	0.62	0.65	0.84	0.43	0.60

RF, Rectus Femoris; VL, Vastus Lateralis; VM, Vastus Medialis; BF, Biceps Femoris; ST, Semitendinosus; LG, Lateral Gastrocnemius; ML, Medial Gastrocnemius

*PDM refers to the method of normalization by using the peak values through ADLs

**PDM refers to the method of normalization by using the peak values through ADLs and MVC.

**Table 4:** Root mean square (RMSD), absolute difference (ABSD), and percentage difference (%D) between the amplitude of knee flexors EMGs normalized using the isometric MVC method and MDM, PDM, *PDM, and **PDM methods.

Knee Flexors													
		BF			ST			LG			MG		
		RMSD	ABSD	%D	RMSD	ABSD	%D	RMSD	ABSD	%D	RMSD	ABSD	%D
**MVC: MDM**	Mean	1.51	1.18	2372.06	2.91	2.30	1537.82	5.23	4.44	939.38	5.72	4.52	889.68
	SD	1.58	1.26	2483.34	5.85	4.56	835.27	10.73	9.59	542.97	13.09	10.28	739.26
**MVC: PDM**	Mean	0.79	0.63	313.96	2.28	1.78	231.89	4.06	3.60	80.68	4.81	3.79	111.04
	SD	2.07	1.65	343.71	6.42	5.02	158.89	11.90	10.57	100.14	14.03	11.02	94.80
**MVC:** *PDM	Mean	0.74	0.59	158.65	2.23	1.74	110.33	4.05	3.58	59.49	4.79	3.77	94.83
	SD	2.09	1.67	237.10	6.44	5.03	78.27	11.90	10.57	49.30	14.03	11.03	84.56
**MVC:** **PDM	Mean	0.71	0.57	58.22	2.16	1.69	34.19	4.02	3.56	29.24	4.74	3.73	40.17
	SD	2.10	1.67	97.65	6.46	5.05	32.30	11.91	10.58	30.72	14.05	11.04	38.27

BF, Biceps Femoris; ST, Semitendinosus; LG, Lateral Gastrocnemius; ML, Medial Gastrocnemius

*PDM refers to the method of normalization by using the peak values through ADLs

**PDM refers to the method of normalization by using the peak values through ADLs and MVC.

**Table 5:** Root mean square (RMSD), absolute difference (ABSD), and percentage difference (%D) between the amplitude of knee extensors EMGs normalized using the isometric MVC method and MDM, PDM, *PDM, and **PDM methods.

Knee Extensors										
		RF			VL			VM		
		RMSD	ABSD	%D	RMSD	ABSD	%D	RMSD	ABSD	%D
**MVC: MDM**	Mean	0.97	0.81	2142.97	2.76	1.94	1826.84	1.43	1.05	2421.68
	SD	0.29	0.25	1885.32	3.77	2.64	2184.82	0.37	0.18	2655.96
**MVC: PDM**	Mean	0.26	0.21	585.63	1.90	1.35	260.08	0.14	0.10	241.95
	SD	0.25	0.21	770.35	4.52	3.17	340.95	0.07	0.05	314.31
MVC: *PDM	Mean	0.12	0.10	103.64	1.86	1.32	83.39	0.08	0.07	67.22
	SD	0.17	0.14	61.40	4.60	3.23	86.81	0.09	0.07	67.19
MVC: **PDM	Mean	0.10	0.09	45.03	1.87	1.33	84.33	0.09	0.07	51.21
	SD	0.18	0.15	35.59	4.60	3.23	88.32	0.09	0.08	40.76

RF, Rectus Femoris; VL, Vastus Lateralis; VM, Vastus Medialis

*PDM refers to the method of normalization by using the peak values through ADLs

**PDM refers to the method of normalization by using the peak values through ADLs and MVC.

## 4. Discussion

Patients with neurological disorder are not always able to perform a maximal effort contraction during MVC recordings. Results of this study indicate that normalization by adopting the peak value measured during different daily activities in addition to MVCs (**PDM) can mimic the relative amplitude expressed when using MVCs alone and therefore overcome this problem. In addition, measuring peak EMG during ADLs is useful to determine the maximum capacity of each muscle required to perform these daily activities during their functional range of motion. In situations where MVC are not feasible, normalization by *PDM has been shown to be superior to normalization using MDM and PDM methods.

As shown in Fig 1, muscle activation patterns did not change across the different methods of normalization. Whereas the relative amplitude, which gives an indication on the strategies that subjects used to activate their muscles during a specific task, are changed among the normalization methods[13]. Moreover, these muscle activations play a role in predicting muscle forces, so it’s important to adopt the method of normalization that reflects the subject’s ability to contract the muscle.

As illustrated in Figs 1 and 2, the EMG of gastrocnemius peaked at approximately 70% of stance phase, after toe off when the knee moves into peak flexion. This muscle acts as an important knee and ankle joint flexor whilst providing joint stability. Whereas, quadriceps activated in early stance, acts with the hamstring muscle to stabilize the knee joint at the weight acceptance phase of the gait cycle.

Fig 1B and 1C, shows normalisation by MDM and PDM respectively and illustrates that these methods cannot be used calculate muscle co-contraction or estimate muscle forces, as the magnitude of the muscle activation does not reflect the true muscle activity. [3] demonstrated that EMG normalized by the peak value through gait cycle “does not indicate the muscle’s ability to activate. Accordingly, the amount of activation cannot be related to any physiological measure and patients’ inability to contract the muscle due to pain inhibition, and altered neuromuscular performance, may not be observed” [14]. As illustrated in Fig 1F, the muscle activity curves resulting from **PDM more closely matches MVC, Fig 1D, in terms of relative amplitude between the three groups of muscles. Fig 1E also shows acceptable matching of *PDM and MVC for the quadriceps and gastrocnemii. However since the hamstrings are activated more highly than the quadriceps, this muscle does not fully mirror the outputs from MVC. This method, although desirable for cohorts where MVCs cannot be obtained, should be interpreted with caution, or other additional activities considered for inclusion to this method.

Different approaches, such as RMSD, ABSD and %D, were used to compare the amplitude as well as the pattern of the EMG normalized by MVC with the MDM, PDM, *PDM, and **PDM (Tables 4 and 5). These are presented in terms of mean and SD for all participants, six stances for each one. By comparing MVC with the other four methods of normalization, **PDM and*PDM show minor differences in the level of activation, especially for rectus femoris (Table 5), the mean RMSD and ABSD for the aforementioned muscle were 0.10, 0.09 0.12, 0.10 respectively. Moreover %D also gives a clear overview of the similarities between MVC and the other methods, and range between (29.24–84.33) in **PDM to (889.68–2421.68) in MDM. This indicates that both *PDM and **PDM would be the closest results compared to the MVC, as illustrated in Table 3.

To enable comparisons with previous literatures [13], CV, VR, RMSD, ABSD, %D were considered. However, a number of studies [1, 11, 25] have just measured CV to demonstrate the intra and inter-individual variability. [16] revealed the limitation of this coefficient to investigate the variability across subjects, and[13, 26] measured VR as well as CV to investigate the variability between subjects that have different means.

Table 1 presents the variability between trials (averaged for 10 subjects). CVs ranged from 0.31, in medial gastrocnemius, to 0.53, in Rectus femoris. There is a good agreement between the CV and VR presented here and those calculated in [13, 25] for Vastus lateralis, Vastus medialis, Biceps femoris and Semitendinosus, with the exception of lower CV for Biceps femoris (0.48 calculated in this study, compared to 0.62 in [25]). A similar trend was seen with VR, which ranged from 0.17 in Medial gastrocnemius to 0.34 in Rectus femoris. In agreement with [13], the intra-individual variability of the un-normalized EMGs was identical to that normalized by different methods of normalization.

In terms of inter-subject variability, the CV and VR values listed in Tables 2 and 3 are similar to the values presented in [11, 13], with the exception of higher inter-individual variability for the MVC method. Other studies [23, 27] have reported that both CV and VR are sensitive to the processing parameters of the EMG signal, such as the cut off frequency of the linear envelope. Therefore smoothing the signal may remove meaningful aspects of the original data and will consequently produce a lower CV and VR.

Regarding the CV values, MVC methods have greater variability between subjects especially for Semitendinosus, 3.92. For the **PDM method, both Rectus femoris and Semitendinosus show a greater variability compared to the other muscles. Across the different methods of normalization the ranking of inter-individual variability between muscles are different. However, this variability arises from those muscles combining efforts in different ways for different individuals to perform level gait. Therefore, the peak values through gait would differ depending on how the subject’s muscles are coordinated and correlated.

Also in agreement with [13, 28] inter-individual VR from the different normalization methods for each muscle showed a similar trend to CV. However, both PDM and MDM through walking reduced inter-individual variability the most, in comparison to the MVC; these methods do not have the potential to provide any information on the degree of muscle activation that occurs during gait. Instead, MVC reflects the true activity in neural drive. Both MVC and **PDM are very similar, in terms of their relative amplitude of quadriceps, hamstrings, and gastrocnemii, however **PDM has lower inter-individual variability.

Fig 2 illustrates the normalization across 10 healthy subjects, presenting their mean and standard deviation. Overall, the relative amplitude illustrated in Fig 2 has almost a similar scenario that seen in Fig 1. The high standard deviation across subjects that can be seen in Fig 2D, MVC method, was due to two participants having lower MVC EMG peak signals for specific muscles compared to their EMG peaks during gait. It can be interpreted that the subjects are able to reach their maximal activity during their functional range of motion, however, when they attempt to maximally contract their muscles outside this range, they fail to reach the maximum value capacity. Additionally, the high standard deviation resulting from the MVC method highlights the negative impact of this method of normalization. If control subjects are unable to elicit maximal isometric contractions; comparisons of activity levels between muscles in different individuals are not valid. This in turn may have bigger negative impact in patient cohorts where they cannot maximally contract.

This work illustrates the higher reliability of **PDM and *PDM than MVC in EMG normalization. The findings from this study have important applications to examiners who apply EMG methodology in diagnosis and evaluation of disease and treatment. Moreover, this study provides alternative approaches to normalization that overcomes the difficulty which may be encountered in securing a maximal contraction from patients with neurological dysfunction; i.e., patients with Osteoarthritis (OA).

Further study is needed to determine if the findings from this study can be applied to other cohorts, i.e., to detect differences in muscle activity between OA and control subjects, to test the sensitivity of the methods recommended.

## 5. Conclusion

Normalization by adopting the peak value through ADLs, **PDM, provided a good ability to reflect the relative magnitude of muscle activity, in a similar way to that seen in MVC and with excellent reliability across subjects. It is also useful for determining on-off timings of muscle activity. Therefore, this method of normalization could be used alternatively when the subject has difficulties with preforming MVCs, i.e. patients with osteoarthritis, or even if their MVCs were less than their muscle activation through walking. If the activities required for **PDM are not available then *PDM is the second preferred option.

### Limitations

The study was conducted on a small number of participants to generate important information that can be applied to a large cohort. The methods of normalization recommended from this study (*PDM and **PDM) have a high coefficient of variation and variance ratio, however, they are still lower than the commonly used MVC.
